# Efeito do esquema 3HP na conclusão do tratamento preventivo da tuberculose em pessoas que vivem com HIV: uma coorte retrospectiva no Brasil

**DOI:** 10.1590/0102-311XPT231024

**Published:** 2025-10-03

**Authors:** João Paulo Cola, Gustavo Silva dos Santos, Fernanda Mattos de Souza, Carolina Maia Martins Sales, Heriederson Sávio Dias Moura, Ricardo Alexandre Arcêncio, Ethel Leonor Noia Maciel, Thiago Nascimento do Prado

**Affiliations:** 1 Laboratório de Epidemiologia, Universidade Federal do Espírito Santo, Vitória, Brasil.; 2 Faculdade MULTIVIX de São Mateus, São Mateus, Brasil.; 3 Programa de Pós-graduação em Clínica Médica, Universidade Federal do Rio de Janeiro, Rio de Janeiro, Brasil.; 4 Departamento de Enfermagem, Universidade Federal do Espírito Santo, Vitória, Brasil.; 5 Escola de Enfermagem de Ribeirão Preto, Universidade de São Paulo, Ribeirão Preto, Brasil.; 6 Programa de Pós-graduação em Saúde Coletiva, Universidade Federal do Espirito Santo, Vitória, Brasil.

**Keywords:** Tuberculose Latente, HIV, Prevenção de Doenças, Adesão à Medicação, Latent Tuberculosis, HIV, Disease Prevention, Medication Adherence, Tuberculosis Latente, VIH, Prevención de Enfermedades, Cumplimiento de la Medicación

## Abstract

As perdas na cascata de cuidados do tratamento preventivo da tuberculose (TB) em pessoas vivendo com HIV/aids (PVHIV) são frequentes, desde a identificação de indivíduos em risco até a conclusão do tratamento. Este estudo analisou os fatores associados à conclusão do tratamento preventivo da TB e o efeito do esquema 3HP na ocorrência desse desfecho nas PVHIV entre 2021 e 2023 no Brasil. Conduziu-se uma coorte retrospectiva com dados secundários do Sistema de Informação para Notificação das Pessoas em Tratamento da Infecção Latente pelo *Mycobacterium tuberculosis* (IL-TB). Incluiuí-se as PVHIV com 18 anos ou mais, independentemente da contagem de células linfócitos T-CD4+, notificadas no IL-TB como caso novo ou reexposição. A conclusão do tratamento preventivo da TB foi o desfecho. Empregou-se regressão de Poisson com variância robusta para calcular o risco relativo de completar o tratamento preventivo da TB, com intervalo de 95% de confiança. Estimou-se o efeito médio do esquema 3HP na conclusão do tratamento preventivo da TB, ponderado por um escore de propensão. Incluiu-se 15.171 PVHIV, sendo que 11.546 (76%) completaram o tratamento preventivo da TB. A conclusão do tratamento preventivo da TB foi maior em indivíduos com idade ≥ 60 anos, com prova tuberculínica ≥ 5mm e que utilizaram o esquema 3HP. O esquema 3HP demonstrou um efeito médio de aumentar em 11% a conclusão do tratamento preventivo da TB. Os resultados evidenciam o efeito positivo do 3HP na conclusão do tratamento preventivo da TB em PVHIV, em comparação com os esquemas de monoterapia com rifampicina ou isoniazida, o que sugere o potencial do 3HP para otimizar as estratégias de prevenção da TB em PVHIV.

## Abstract (en)

Losses in the tuberculosis (TB) preventive treatment cascade among people living with HIV/AIDS (PLHIV) are frequent, ranging from the identification of individuals at risk to treatment completion. This study aimed to analyze the factors associated with the completion of TB preventive treatment and the effect of the 3HP regimen on this outcome among PLHIV in Brazil between 2021 and 2023. A retrospective cohort study was conducted using secondary data from the Information System for the Notification of People Undergoing Treatment for Latent *Mycobacterium tuberculosis* Infection (IL-TB). PLHIV aged 18 years or older, regardless of CD4+ T-cell count, who were reported to IL-TB as new cases or re-exposure, were included. The outcome was the completion of TB preventive treatment. Poisson regression with robust variance was used to estimate the relative risk of treatment completion, with a 95% confidence interval. The average effect of the 3HP regimen on treatment completion was estimated using propensity score weighting. A total of 15,171 PLHIV were included, of whom 11,546 (76%) completed TB preventive treatment. Completion rates were higher among individuals aged ≥60 years, those with tuberculin skin test results ≥5 mm, and those who received the 3HP regimen. The 3HP regimen showed an average effect of increasing TB preventive treatment completion by 11%. These findings highlight the positive impact of the 3HP regimen compared to monotherapy with rifampin or isoniazid, suggesting its potential to enhance TB prevention strategies among PLHIV.

## Abstract (es)

Las pérdidas en la cascada de atención del tratamiento preventivo de la tuberculosis (TB) en personas que viven con VIH/sida (PVVIH) son frecuentes, desde la identificación de individuos en riesgo hasta la finalización del tratamiento. Este estudio analizó los factores asociados con la finalización del tratamiento preventivo de la TB y el efecto del esquema 3HP en dicho desenlace entre las PVVIH en Brasil, entre 2021 y 2023. Se llevó a cabo una cohorte retrospectiva utilizando datos secundarios del Sistema de Información para la Notificación de Personas en Tratamiento por Infección Latente por *Mycobacterium tuberculosis* (IL-TB). Se incluyeron PVVIH de 18 años o más, independientemente del recuento de linfocitos T CD4+, notificadas en el IL-TB como caso nuevo o por reexposición. El desenlace fue la finalización del tratamiento preventivo de la TB. Se utilizó regresión de Poisson con varianza robusta para estimar el riesgo relativo de completar el tratamiento, con un intervalo de confianza del 95%. El efecto promedio del esquema 3HP en la finalización del tratamiento se estimó mediante un puntaje de propensión ponderado. Se incluyeron 15.171 PVVIH, de las cuales 11.546 (76%) completaron el tratamiento preventivo de la TB. La finalización del tratamiento fue mayor en individuos de 60 años o más, con prueba tuberculínica ≥ 5 mm y que utilizaron el esquema 3HP. El esquema 3HP mostró un efecto promedio de aumento del 11% en la finalización del tratamiento preventivo. Los resultados evidencian el efecto positivo del 3HP en comparación con esquemas de monoterapia con rifampicina o isoniacida, lo que sugiere su potencial para optimizar las estrategias de prevención de la TB en PVVIH.

## Introdução

O *Mycobacterium tuberculosis* é a principal causa de morte entre as pessoas que vivem com HIV/aids (PVHIV) ^1^. A infecção pelo HIV compromete o sistema imunológico, o que reduz sua efetividade contra patógenos e aumenta significativamente o risco de adoecimento por tuberculose (TB) ^2^ ^,^ ^3^. Dessa forma, o tratamento preventivo da TB emerge como uma estratégia efetiva para reduzir a morbimortalidade por TB entre as PVHIV ^1^ ^,^ ^2^ ^,^ ^3^.

O Brasil realiza esforços para ampliar a cobertura do tratamento preventivo da TB. Desde 2011, o tratamento preventivo da TB é recomendado para as PVHIV com idade maior ou igual a 18 anos com prova tuberculínica positiva (PT ≥ 5mm) e radiografia de tórax normal ^4^. Em 2014, o Ministério da Saúde recomendou a realização do tratamento preventivo da TB em PVHIV com contagem de linfócitos T-CD4+ menor ou igual a 350células/mm^3^ em caso de indisponibilidade da PT. Em seguida, recomendou a realização do tratamento preventivo da TB nesse grupo populacional independentemente da realização de teste diagnóstico para infecção por TB (ITB). Ambas as recomendações com indicação de implementação a nível individual após afastado diagnóstico de TB. O QuantiFERON-TB Gold Plus test (QFT-Plus, https://www.qiagen.com/br), um dos IGRA (sigla em inglês para *interferon gamma release assay*), foi incorporado ao Sistema Único de Saúde (SUS) no final de 2020 para o diagnóstico de infecção por TB nas PVHIV e em outros grupos-alvo para o enfrentamento da TB ^4^ ^,^ ^5^. Ademais, o Ministério da Saúde incorporou novos regimes de tratamento preventivo da TB, como o esquema de doses semanais de isoniazida associada à rifapentina durante três meses (3HP), incorporado em 2021 ^4^ ^,^ ^5^.

Em 2018, o Ministério da Saúde implantou o Sistema de Informação para Notificação das Pessoas em Tratamento da Infecção Latente pelo *M. tuberculosis* (IL-TB) para promover a vigilância da infecção por TB ^5^. Desde a implantação desse sistema, entre 2018 e 2023, observou-se uma tendência crescente no número de pessoas notificadas que iniciaram o tratamento preventivo da TB no país, totalizando 163.885 notificações ^5^ ^,^ ^6^. As PVHIV estão no segundo grupo com maior número de registros ^5^ ^,^ ^6^. Além disso, o número de notificações para o esquema 3HP ultrapassou os demais esquemas terapêuticos, atingindo 59,9% do total ^5^ ^,^ ^6^.

Embora as ações de expansão do tratamento preventivo da TB apresentem avanços, a conclusão do tratamento é um desafio ^3^ ^,^ ^5^ ^,^ ^7^. Um estudo brasileiro de coorte retrospectiva, que analisou os dados de notificação de tratamento preventivo da TB no IL-TB entre 2018 e 2020, demonstrou que pessoas em situações de vulnerabilidade social e financeira, assim como aqueles em regimes de tratamento preventivo da TB mais longos, tiveram maior probabilidade de não concluírem o tratamento ^7^. 

Por meio de uma revisão da literatura, pode-se observar poucas evidências em contexto nacional sobre o impacto do esquema 3HP na conclusão do tratamento preventivo da TB entre PVHIV, especialmente após sua incorporação recente no SUS ^8^. Pouco se sabe sobre os fatores que influenciam a conclusão do tratamento preventivo da TB especificamente entre PVHIV no Brasil após a expansão do 3HP e a implantação do IL-TB.

Nesse sentido, o estudo teve como objetivo analisar os fatores associados à conclusão do tratamento preventivo da TB e o efeito do esquema 3HP na ocorrência desse desfecho nas PVHIV entre 2021 e 2023 no Brasil.

## Métodos

### Desenho e fonte de dados

Trata-se de uma coorte retrospectiva baseada em dados secundários do IL-TB, que visa monitorar e melhorar a implementação do tratamento preventivo da TB no país. O IL-TB contém dados sociodemográficos, resultados de testes para diagnóstico da infecção por TB, resultados de radiografia de tórax, indicação para tratamento preventivo da TB, condições de saúde e acompanhamento clínico. Desde 2018, o Ministério da Saúde recomenda a notificação de todos os casos que iniciaram o tratamento preventivo da TB nesse sistema de informação ^4^.

No Brasil, as recomendações nacionais orientam o diagnóstico da infecção por TB com base em um resultado positivo da PT ou um IGRA reagente. Após essa etapa, realizam-se avaliação clínica e radiografia de tórax para descartar a presença de TB ^4^.

Para as PVHIV com contagem de linfócitos T-CD4+ ≤ 350células/µL, o tratamento preventivo da TB deve ser iniciado após descartar TB, independentemente da realização de teste diagnóstico para infecção por TB. Para aquelas com contagem de linfócitos T-CD4+ desconhecida ou > 350células/µL, o tratamento preventivo da TB é indicado somente após a confirmação por um teste diagnóstico positivo, seja PT ou IGRA. Desde 2021, o Brasil incorporou o esquema terapêutico 3HP, além dos outros três esquemas já disponíveis nos serviços: a monoterapia com isoniazida por 9 meses (9H), a monoterapia com isoniazida por 6 meses (6H) e a monoterapia com rifampicina por 4 meses (4R) ^4^. 

Todos os esquemas terapêuticos incorporados no Brasil são indicados para a PVHIV. Contudo, o esquema 4R é a primeira escolha para pessoas com mais de 50 anos, com comprometimento hepático, contatos com pessoas com TB monorresistente e intolerância à isoniazida ^4^. Os serviços de saúde acompanham as pessoas que iniciam o tratamento preventivo da TB por meio de consultas regulares em intervalos de 30 ou 60 dias a depender do risco de hepatotoxicidade e da adesão ao tratamento ^4^.

### Critérios de inclusão e exclusão

Adotou-se como critério de inclusão, PVHIV com 18 anos ou mais, independentemente da contagem de células linfócitos T-CD4+, notificadas no IL-TB como caso novo ou reexposição, que iniciaram o tratamento preventivo da TB entre 6 de Julho de 2021 e 31 de Dezembro de 2023. Excluiu-se pessoas: que tiveram tratamento interrompido devido a reação adversa; cujo cuidado foi transferido para outro serviço; que evoluíram para TB; óbitos; cujo tratamento foi interrompido por critérios clínicos; e que não possuíam informação de encerramento do tratamento preventivo da TB.

### Desfecho

O desfecho estudado foi a conclusão do tratamento preventivo da TB. Classificamos as pessoas como tratamento concluído conforme o número de doses necessárias para conclusão do tratamento preventivo da TB de acordo com os diferentes esquemas terapêuticos: 6H, 9H, 4R ou 3HP. 

Para o esquema 3HP, a administração de 12 doses durante 12 a 15 semanas define a conclusão; o esquema 9H necessita de 270 doses ao longo de 9 a 12 meses para completar o tratamento; o esquema 6H requer 180 doses entre 6 e 9 meses; e o esquema 4R demanda 120 doses entre 4 e 6 meses ^4^.

### Variáveis

Analisaram-se as seguintes variáveis de exposição: sexo, idade (categorizada em faixas etária 18-20, 20-39, 40-59, e ≥ 60 anos), raça/cor da pele, ser contato de pessoas com TB, cicatriz da vacina BCG (Bacilli Calmette-Guérin), radiografia de tórax, resultado da PT ou do IGRA e esquema terapêutico usado no tratamento preventivo da TB. 

Para analisar o efeito do 3HP na conclusão do tratamento, classificamos as pessoas em dois grupos: os expostos, definido como aqueles que utilizaram o 3HP, e os não expostos, aqueles que receberam os demais esquemas terapêuticos (6H, 9H ou 4R).

### Análises

Para a análise descritiva, calcularam-se as frequências absolutas (N) e relativas (%) das variáveis sociodemográficas e clínicas, estratificadas pela conclusão do tratamento preventivo da TB. Empregou-se o teste qui-quadrado de Pearson para analisar as diferenças entre os grupos. 

Posteriormente, empregou-se a regressão de Poisson com variância robusta para calcular o risco relativo (RR) de completar o tratamento preventivo da TB, com intervalo de 95% de confiança (IC95%). As variáveis que apresentaram valor de p ≤ 0,20 na análise descritiva foram incluídas em modelo bruto. O modelo final foi ajustado pelas variáveis que apresentaram valor de p ≤ 0,05 na regressão de Poisson bruta.

Para analisar o efeito do 3HP na conclusão do tratamento preventivo da TB, empregou-se o teste qui-quadrado de Pearson para avaliar a associação entre a conclusão do tratamento preventivo da TB entre o esquema 3HP em comparação com os outros esquemas. Para garantir o balanceamento e permutabilidade dos grupos em relação à exposição (tratamento preventivo da TB com 3HP), estimou-se um escore de propensão que indica a probabilidade condicional de receber o esquema 3HP em função das variáveis ​​preditoras observadas ^9^ ^,^ ^10^. 

O escore de propensão foi estimado por um modelo logístico, no qual ter realizado o tratamento preventivo da TB com 3HP foi a variável dependente, incluindo as seguintes variáveis preditoras: idade, sexo, raça/cor da pele e ser contato de pessoas com TB ^7^. Após, calculou-se a ponderação pelo escore de propensão, utilizando a ponderação inversa da probabilidade do escore de propensão ^9^ ^,^ ^10^ ^,^ ^11^. Por meio desse método, permitiu-se a criação de uma pseudopopulação contrafactual balanceada. O balanceamento de variáveis foi considerado adequado quando a diferença padronizada absoluta das médias foi < 0,10 e o desvio padrão (DP) e a razão de variância entre 0,80 e 1,20. 

O risco de completar o tratamento preventivo da TB com o esquema 3HP foi estimado pela regressão de Poisson de variância robusta, ponderada pela ponderação pelo escore de propensão. Os resultados foram expressos em RR com IC95%. Em seguida, calculou-se o efeito médio do esquema 3HP na conclusão do tratamento, ponderado pela ponderação pelo escore de propensão. 

Todas as análises foram realizadas no software Stata v. 14.0 (https://www.stata.com), utilizando os comandos *logit*, *glm family* (poisson) *robust*, *pscore*, *teffects*, *tebalance*.

### Aspectos éticos

A pesquisa foi aprovada pelo Comitê de Ética em Pesquisa do Centro de Ciências da Saúde da Universidade Federal do Espírito Santo (CAAE: 79099524.4.0000.5060). Os dados foram disponibilizados pelo Coordenação Geral de Vigilância de Doenças Respiratórias Transmissíveis com Condições Crônicas, Departamento de Doenças com Condições Crônicas e Infecções Sexualmente Transmissíveis, Secretaria de Vigilância em Saúde, Ministério da Saúde.

## Resultados

**Figura 1:**
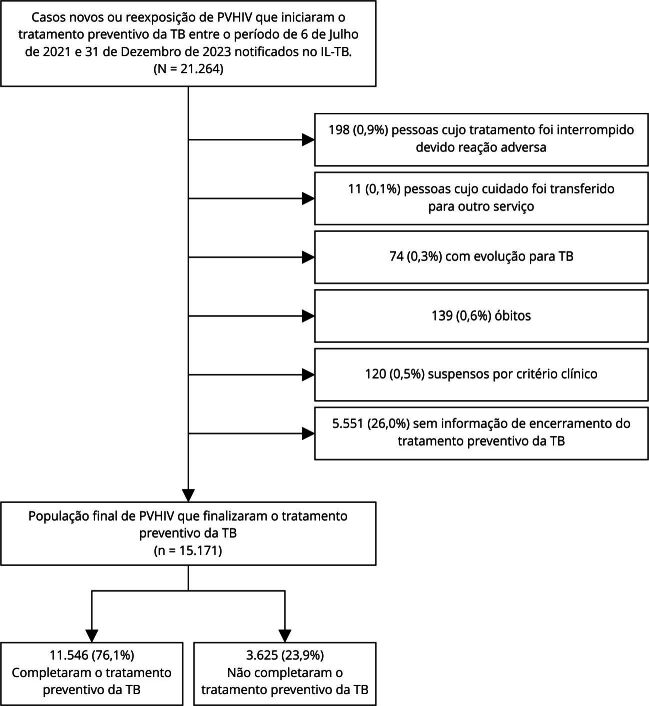
Pessoas que vivem com HIV/aids (PVHIV) notificadas como caso novo ou reexposição no Sistema de Informação para Notificação das Pessoas em Tratamento da Infecção Latente pelo *Mycobacterium tuberculosis* (IL-TB), entre 6 de julho de 2021 e 31 de dezembro de 2023, no Brasil incluídas no estudo.

Entre o período de 6 de julho de 2021 e 31 de dezembro de 2023, notificaram-se 21.264 casos novos ou reexposição de PVHIV que iniciaram o tratamento preventivo da TB. Foram excluídas 198 (0,9%) pessoas cujo tratamento foi interrompido devido a reação adversa, 11 (0,1%) cujo cuidado foi transferido para outro serviço, 74 (0,3%) com evolução para TB, 139 (0,6%) óbitos, 120 (0,5%) suspensos por critérios clínicos e 5.551 (26%) sem informação de encerramento do tratamento preventivo da TB. A população final compreendeu 15.171 PVHIV, sendo que 11.546 (76,1%) completaram o tratamento (Figura 1).

TB: tuberculose.

A Tabela 1 apresenta a distribuição das características sociodemográficas e clínicas das PVHIV conforme a conclusão do tratamento preventivo da TB. Observou-se maior proporção de conclusão do tratamento preventivo da TB entre indivíduos com idade superior a 60 anos (81,8%) e do sexo masculino (76,7%). Não se verificaram diferenças significativas na conclusão do tratamento preventivo da TB em relação à raça/cor da pele e ao histórico de contato de pessoas com TB. A vacinação com o BCG (77,9%), resultado positivo na PT (≥ 5mm) (80,7%) e os esquemas terapêuticos 3HP (84,5%) e 4R (85,2%) também exibiram as maiores frequências de conclusão do tratamento preventivo da TB.

**Tabela 1:** Distribuição das características sociodemográficas e clínicas das pessoas que vivem com HIV/aids (PVHIV) de acordo com a conclusão do tratamento preventivo da tuberculose (TB), entre 6 de julho de 2021 e 31 de dezembro de 2023, Brasil.

Variáveis	Tratamento preventivo da TB		Total n (%)	Valor de p *
	Incompleto n (%)	Completo n (%)		
Faixa etária (anos)				< 0,001
< 20	41 (29,7)	97 (70,2)	138 (100,0)	
20-39	1.932 (26,5)	5.359 (73,5)	7.291 (100,0)	
40-59	1.410 (21,9)	5.002 (78,0)	6.412 (100,0)	
≥ 60	2.42 (18,2)	1.088 (81,8)	1.330 (100,0)	
Sexo				0,005
Feminino	1.117 (25,4)	3.276 (74,5)	4.393 (100,0)	
Masculino	2.508 (23,2)	8.270 (76,7)	10.778 (100,0)	
Raça/Cor da pele				0,156
Branca	1.122 (23,2)	3.698 (76,7)	4.820 (100,0)	
Preta/Parda	2.196 (24,2)	6.843 (75,7)	9.039 (100,0)	
Amarelos/Indígenas	42 (21,0)	158 (79,0)	200 (100,0)	
Ser contato de pessoas com TB				0,138
Não/Não sabe	3.414 (23,9)	10.833 (76,0)	14.247 (100,0)	
Sim	211 (22,8)	713 (77,1)	924 (100,0)	
Vacinado com BCG				< 0,001
Não/Não sabe	1.713 (26,3)	4.800 (73,7)	6.513 (100,0)	
Sim	1.912 (22,0)	6,746 (77,9)	8.658 (100,0)	
Radiografia de tórax				0,091
Sem alteração	2.802 (23,0)	9.382 (77,0)	12.184 (100,0)	
Alteração não sugestiva de TB	196 (25,6)	568 (74,3)	764 (100,0)	
IGRA				< 0,001
Negativo	14 (14,5)	82 (85,4)	96 (100,0)	
Positivo	183 (15,0)	1.033 (84,9)	1.216 (100,0)	
Indeterminado	13 (26,5)	36 (73,4)	49 (100,0)	
Não realizado	3.415 (24,7)	10.395 (75,2)	13.810 (100,0)	
Resultado da PT				< 0,001
< 5mm	437 (24,7)	1.331 (75,2)	1.768 (100,0)	
≥ 5mm	970 (19,2)	4.058 (80,7)	5.028 (100,0)	
Esquema terapêutico				< 0,001
Monoterapia com INH (6H/9H)	2.896 (27,6)	7.562 (72,3)	10.458 (100,0)	
4R	28 (14,7)	162 (85,2)	190 (100,0)	
3HP	701 (15,5)	3.822 (84,5)	4.523 (100,0)	

3HP: 12 semanas de rifapentina com isoniazida; 4R: 4 meses de rifampicina; IGRA: sigla em inglês para *interferon gamma release assay*; INH: isoniazida; PT: prova tuberculínica.

* Qui-quadrado de Pearson.

Na análise bruta, a conclusão do tratamento preventivo da TB entre as PVHIV demonstrou associação com idade ≥ 60 anos, sexo masculino, vacinação com o BCG, PT ≥ 5mm e utilização dos esquemas terapêuticos 4R e 3HP. Na análise ajustada, observou-se maior risco de completar o tratamento preventivo da TB entre indivíduos com idade ≥ 60 anos (RR = 1,19; IC95%: 1,01-1,41), com PT ≥ 5mm (RR = 1,07; IC95%: 1,04-1,10) e que utilizaram o esquema terapêutico 3HP (RR = 1,13; IC95%: 1,10-1,15) (Tabela 2).

**Tabela 2:** Razões de risco da conclusão do tratamento preventivo da tuberculose (TB), segundo características sociodemográficas e clínicas das pessoas que vivem com HIV/aids (PVHIV) e que iniciaram o tratamento, entre 6 de julho de 2021 e 31 de dezembro de 2023, Brasil.

Variáveis	Análise bruta		Análise ajustada	
	RR (IC95%)	Valor de p	RR (IC95%)	Valor de p
Faixa etária (anos)		< 0,001		< 0,001
< 20	1,00		1,00	
20-39	1,04 (0,93-1,16)		1,05 (0,89-1,24)	
40-59	1,10 (0,99-1,23)		1,14 (0,97-1,34)	
≥ 60	1,16 (1,04-1,30)		1,19 (1,01-1,41)	
Sexo		0,005		0,105
Feminino	1,00		1,00	
Masculino	1,02 (1,01-1,04)		1,02 (0,99-1,05)	
Raça/Cor da pele		0,238		-
Branca	1,00		-	
Preta/Parda	0,98 (0,96-1,00)		-	
Amarelos/Indígenas	1,02 (0,95-1,10)		-	
Ser contato de pessoas com TB		0,426		-
Não/Não sabe	1,00		-	
Sim	1,01 (0,97-1,05)		-	
Vacinado com BCG		< 0,001		0,756
Não/Não sabe	1,00		1,00	
Sim	1,05 (1,03-1,07)		0,99 (0,97-1,02)	
Radiografia de tórax		0,107		-
Sem alteração	1,00		-	
Alteração não sugestiva de TB	0,96 (0,92-1,00)		-	
Resultado da PT		< 0,001		< 0,001
< 5mm	1,00		1,00	
≥ 5mm	1,07 (1,04-1,10)		1,07 (1,04-1,10)	
Esquema terapêutico		< 0,001		< 0,001
Monoterapia com INH (6H/9H)	1,00		1,00	
4R	1,17 (1,11-1,25)		1,07 (0,99-1,15)	
3HP	1,16 (1,14-1,18)		1,13 (1,10-1,15)	

3HP: 12 semanas de rifapentina com isoniazida; 4R: 4 meses de rifampicina; IC95%: intervalo de 95% de confiança; INH: isoniazida; RR: risco relativo; PT: prova tuberculínica.

A Tabela 3 descreve o perfil sociodemográfico e clínico das PVHIV que utilizaram o esquema 3HP. A utilização deste esquema terapêutico foi mais frequente entre indivíduos na faixa etária de 20-59 anos (20-39 anos: 48,8%; 40-59 anos: 41,2%), do sexo masculino (72,1%), com raça/cor da pele preta ou parda (68,7%), sem histórico conhecido de contato de pessoas com TB ou com informação ignorada sobre o contato (92,5%), vacinados com o BCG (64,9%) e com resultado da PT ≥ 5mm (73%).

**Tabela 3:** Distribuição das características sociodemográficas e clínicas das 15.171 pessoas que vivem com HIV/aids (PVHIV) e que iniciaram o tratamento preventivo da tuberculose (TB) com o esquema 3HP, entre 6 de julho de 2021 e 31 de dezembro de 2023, Brasil.

Variáveis	Esquema terapêutico do tratamento preventivo da TB	
	3HP n (%)	Outros * n (%)
Faixa etária (anos)		
< 20	35 (0,7)	103 (0,9)
20-39	2.208 (48,8)	5.083 (47,7)
40-59	1.866 (41,2)	4.546 (42,6)
≥ 60	414 (9,1)	916 (8,6)
Sexo		
Feminino	1.258 (27,8)	3.135 (29,4)
Masculino	3.265 (72,1)	7.513 (70,5)
Raça/Cor da pele		
Branca	1.296 (29,9)	3.524 (36,2)
Preta/Parda	2.974 (68,7)	6.065 (62,3)
Amarelos/Indígenas	59 (1,3)	141 (1,4)
Ser contato de pessoas com TB		
Não/Não sabe	4.187 (92,5)	10.060 (94,4)
Sim	336 (7,4)	588 (5,5)
Vacinado com BCG		
Não/Não sabe	1.584 (35,0)	4.929 (46,2)
Sim	2.939 (64,9)	5.719 (53,7)
Radiografia de tórax		
Sem alteração	3.647 (93,0)	8.537 (94,5)
Alteração não sugestiva de TB	272 (6,9)	492 (5,4)
Resultado da PT		
< 5mm	472 (27,0)	1.296 (25,6)
≥ 5mm	1.276 (73,0)	3.752 (74,3)

3HP: 12 semanas de rifapentina com isoniazida; PT: prova tuberculínica.

* Monoterapia com isoniazida ou 4 meses de rifampicina.

O esquema 3HP (84,5%) demonstrou maior taxa de conclusão do tratamento preventivo da TB em comparação com os demais esquemas terapêuticos (72,5%). Na análise ajustada pela ponderação pelo escore de propensão, o 3HP associou-se a uma maior conclusão do tratamento preventivo da TB. O risco de completar o tratamento preventivo da TB com o esquema 3HP foi 1,15 vezes maior (IC95%: 1,13-1,19) em relação aos outros esquemas terapêuticos. O efeito médio da utilização do 3HP, ponderado pelo escore de propensão, resultou em uma melhora de 11% (coeficiente 0,11 − IC95%: 0,10-0,13) na conclusão do tratamento preventivo da TB entre PVHIV (Tabela 4).

**Tabela 4:** Distribuição e estimativas do efeito do esquema 3HP na completude do tratamento preventivo da tuberculose (TB) em pessoas que vivem com HIV e que iniciaram o tratamento preventivo da TB, entre 6 de julho de 2021 e 31 de dezembro de 2023, Brasil.

Esquema terapêutico	Tratamento preventivo da TB		Valor de p *
	Completo n (%)	Incompleto n (%)	
3HP	3.822 (84,5)	701 (15,5)	< 0,001
Outros **	7.724 (72,5)	2.924 (27,4)	
Total	11.546 (76,1)	3.625 (23,8)	
Regressão de Poisson			
Análise não ajustada [RR (IC95%)]	1,16 (1,14-1,18)		
Análise ajustada *** [RR (IC95%)]	1,15 (1,13-1,19)		
ATE ^#^ [coeficiente (IC95%)]	0,11 (0,10-0,13)		

3HP: 12 semanas de rifapentina com isoniazida; coeficiente: diferença dos efeitos para o esquema 3HP e outros esquemas; IC95%: intervalo de 95% de confiança.

* Teste qui-quadrado de Pearson;

** Monoterapia com isoniazida ou 4 meses de rifampicina;

*** Modelo ponderado pelo escore de propensão;

^#^ Efeito médio do tratamento ponderado pelo escore de propensão.

## Discussão

Os resultados do estudo demonstraram que a utilização do esquema 3HP, idade ≥ 60 anos e ter resultado ≥ 5mm na PT foram associados à conclusão do tratamento preventivo da TB entre as PVHIV no Brasil. Em nosso estudo, o esquema 3HP apresentou efeito positivo em comparação com os esquemas de monoterapia com rifampicina ou isoniazida em situação pragmática, mesmo após controle de potenciais confundidores. 

Um ensaio clínico randomizado com PVHIV comparou a efetividade e a segurança de um regime de tratamento preventivo da TB com duração de um mês, utilizando rifapentina mais isoniazida com dose diária, em relação ao esquema 9H. Apresentaram-se taxas de conclusão de 97% e 90%, respectivamente, representando uma diferença de 7% entre os esquemas analisados ^12^. Esse resultado assemelha-se aos achados de outro ensaio clínico com a população que não vive com HIV, com os mesmos esquemas terapêuticos analisados ^13^ ^,^ ^14^ ^,^ ^15^. Além da maior frequência de conclusão do tratamento preventivo da TB, o regime encurtado também apresentou maior razão de custo-efetividade nas PVHIV ^16^ ^,^ ^17^ ^,^ ^18^ ^,^ ^19^. 

No Brasil, em vista de melhorar a adesão ao tratamento preventivo da TB e minimizar os erros na tomada dos medicamentos utilizados em doses isoladas, o Ministério da Saúde iniciou no ano de 2024 a distribuição do medicamento rifapentina mais isoniazida comprimido em dose fixa combinada (300/300mg) para o tratamento preventivo da TB. Contudo, mesmo com um esquema encurtado, como o 3HP, a tomada de múltiplos comprimidos em uma única dose e a ocorrência de eventos adversos pode justificar a não conclusão do tratamento preventivo da TB ^20^ ^,^ ^21^ ^,^ ^22^. A incorporação de novos esquemas que diminuam a quantidade de comprimido se faz necessário para avançar na redução das taxas da não conclusão do tratamento preventivo da TB na PVHIV ^3^ ^,^ ^22^ ^,^ ^23^. 

Pessoas idosas foram associadas a uma maior conclusão do tratamento preventivo da TB. As particularidades biopsicossociais inerentes ao processo de envelhecimento diferenciam as pessoas idosas da população adulta, com heterogeneidade observada mesmo dentro dessa faixa etária ^24^ ^,^ ^25^ ^,^ ^26^. A reativação da infecção por TB é considerada a causa principal de adoecimento por TB em pessoas idosas, possivelmente devido à imunossenescência associada ao envelhecimento ^24^ ^,^ ^25^ ^,^ ^26^. As comorbidades, por exemplo, podem proteger em relação à vulnerabilidade de pessoas idosas por, em alguns casos, estarem associadas a uma menor chance de perda de seguimento devido ao acompanhamento regular e à proximidade de serviços de saúde ^27^ ^,^ ^28^. Ao constatar que as pessoas idosas apresentam maior probabilidade de concluir o tratamento preventivo da TB, nossos achados sugerem que o tratamento da infecção por TB pode ser bem tolerado por indivíduos com 60 anos ou mais.

Além disso, constatou-se maior risco de conclusão do tratamento preventivo da TB entre as PVHIV com resultado ≥ 5mm na PT. Uma revisão sistemática, ao analisar a cascata de cuidado da infecção por TB em PVHIV, identificou que a realização de testes diagnósticos para a infecção por TB nessa população associou-se a uma maior proporção de indivíduos que iniciaram e concluíram o tratamento preventivo da TB em países de baixa e média renda ^8^.

Considerando as diversas opções de esquemas terapêuticos para a realização do tratamento preventivo da TB disponíveis no SUS, a escolha deve ser individualizada. Ao iniciar o tratamento preventivo da TB em PVHIV, torna-se imperativo avaliar os benefícios, em termos de prevenção da TB e da morbimortalidade, assim como os potenciais danos, incluindo eventos adversos, interações medicamentosas com os antirretrovirais, a aceitabilidade do tratamento pelos indivíduos, a presença de comorbidades e as condições de vida da pessoa com infecção por TB, o que assegura a continuidade do cuidado longitudinal da PVHIV ^3^. 

Os achados do estudo estão baseados em dados secundários do IL-TB. As notificações no IL-TB são realizadas no início do tratamento e o banco de dados é atualizado durante o acompanhamento, o que possibilita a busca ativa das pessoas que não comparecem às consultas. Em consonância com as recomendações nacionais, as consultas de acompanhamento devem ocorrer em um intervalo máximo de 60 dias ^4^. Esse cenário pode ter contribuído para as taxas de conclusão observadas. 

O estudo apresenta algumas limitações que devem ser consideradas. A notificação do tratamento preventivo da TB no IL-TB não é obrigatória, o que pode ter ocasionado a subnotificação. Deve-se ponderar também que durante o período analisado, o esquema 3HP estava em fase de introdução no Brasil com a realização de treinamentos conduzidos pelo Ministério da Saúde com o objetivo de estabelecer esse regime terapêutico como o preferencial para o tratamento preventivo da TB. Dessa forma, a maior completude observada com o 3HP pode estar relacionada também a uma implementação mais efetiva do tratamento preventivo da TB na prática clínica nos serviços que receberam prioridade para a adoção do 3HP ^5^. No entanto, considera-se que isso não afeta nossos resultados, pois as notificações foram distribuídas por todo o país, bem como o balanceamento da população obtido pela ponderação pelo escore de propensão ao considerar como variáveis preditoras da conclusão do tratamento preventivo da TB com o 3HP a idade, sexo, raça/cor da pele e ser contato de pessoas com TB. A definição de conclusão do tratamento preventivo da TB pode ter sido inconsistente, pois foi registrada por diferentes profissionais de saúde em todo o país. Contudo, os dados foram monitorados e submetidos a uma sequência hierárquica de avaliação da qualidade dos dados. Outro ponto, é que o IL-TB não possui dados adicionais de fatores que podem afetar a conclusão do tratamento preventivo da TB como o uso de álcool e outras drogas, tabagismo, comorbidades e situação de moradia. 

Para estudos futuros, seria oportuno um *linkage* entre banco de dados, e assim a inclusão dessas variáveis, para melhor ajuste dos modelos, e assim análises adicionais estratificadas por situação de vulnerabilidade, para identificar possíveis desigualdades na conclusão do tratamento preventivo da TB.

Os achados do presente estudo indicam a importância de direcionar esforços para a implementação de políticas de saúde que incentivem a utilização do esquema 3HP entre as PVHIV e avaliem a segurança deste esquema em pessoas idosas. Tais políticas podem incluir campanhas de conscientização para o fortalecimento do diagnóstico da infecções por TB, qualificação para os profissionais de saúde e acompanhamento contínuo para garantir a adesão ao tratamento. Ao priorizar o tratamento preventivo da TB com os esquemas reduzidos, é possível não apenas aumentar as taxas de conclusão, mas também contribuir significativamente para a eliminação da TB, prioridade do Programa Brasil Saudável, lançado em 2024 e que visa a eliminação de doenças determinadas socialmente no Brasil.
